# Medical Society of Stark Co. Ohio

**Published:** 1840

**Authors:** 


### MEDICAL SOCIETY OF STARK COUNTY, OHIO.

We hail with pleasure every associated effort of our brethren, for the *principle* of voluntary concert is correct. We have before us the proceedings of the physicians of one of the central counties of Ohio, at a meeting held on the 27th October last, in Canton. A Society was formed, and a code of ethics adopted; from which we extract, with unalloyed gratification, the following rule of professional duty:

"Physicians should never neglect an opportunity of fortifying and promoting the good resolutions of patients suffering under the effects of intemperate lives, and vicious conduct: and, in order that their counsels and remonstrances may have due weight, it will be readily seen, that they should have full claim to the blameless life and high moral character, which we have stated to be a necessary requisite to an honorable stand in the profession."

The spontaneous adoption of such an obligation, is evidence of sound moral feelings, and prevalent sobriety among the gentlemen composing the profession of that county. But justice requires us to say, that in these respects they are not preeminent, as it is unquestionably true, that throughout the entire west, the temperance and general morality of our physicians, are on the increase. Drunkenness, profanity, gambling and infidel boastings, are fast falling into discredit, and seem likely, at no distant time, to be regarded as utterly unprofessional, and even infamous. All the effects of this reformation will be salutary; for an increased devotion to *intellectual* improvement, can never fail to ensue, upon the repudiation of dissolute amusements.

The officers elected at the first meeting of the society were Dr. Whiting, President; Dr. Wallace, Secretary; Dr. Dolbear, Treasurer, and Doctors Estep, Preston, Brockenbush, Dolwigh and Bowen, Censors. D.

